# Civil Hospitals and Military Officials

**Published:** 1915-01-30

**Authors:** 


### The Hospital

The Workers' Newspaper of Administrative Medicine and Institutional Life, Administration, National Insurance and Health.

No. 1493, Vol. LVII. SATURDAY, JANUARY 30, 1915. PRICE ONE PENNY.

### CIVIL HOSPITALS AND MILITARY OFFICIALS.

A painful and unhappy incident is reported from the Longford County Infirmary. It appears that on December 13 last Sir Thomas Myles, who has been appointed Surgeon to the Forces in Ireland, went to the infirmary in order to perform an operation for appendicitis on a soldier, and that Dr. Mayne, who is in charge of the infirmary, refused to grant the use of the operating theatre unless he himself performed the operation. Thereupon the patient was taken by motor-car to Dublin, where he was operated on at the Richmond Hospital. Subsequently the event came before the infirmary committee in the shape of a complaint from the Earl of Granard, K.P., colonel in command of a battalion of the Royal Irish Regiment stationed at Longford, and presumably, therefore, responsible, in a military sense, for the soldier whose appendicitis led to the above unfortunate dispute. After debate, the infirmary committee decided that Dr. Mayne's action was indefensible, and, by a majority, a resolution was carried dismissing him from the control and charge of the hospital. Obviously the incident has public as well as professional aspects. The issue is not so simple as many may be likely to consider it, and a careful study of the facts is necessary if an impartial judgment is to be secured. Here we make no pretence to any knowledge of the occurrence other than can be learned from the public prints, and it is to questions of principle alone that we propose to address ourselves.

When two medical practitioners fall into open controversy on some point of procedure, the general judgment is that the quarrel in essence is a dispute about the interpretation of some authoritative and well-defined code of rules known as professional etiquette. This will no doubt be the public impression in the present case. As a matter of fact, there are no formal laws governing professional relations, but only certain principles which it is necessary to recognise in the interests not of doctors only but also of patients. Now the first of these principles is that the welfare of the sick man is the first consideration. In the instance here under discussion it is certain that this principle was neglected, for an unnecessary journey in a motor-car is not a proceeding likely to promote the welfare of a patient suffering from appendicitis. Rather, therefore, than subject the soldier to such a risk, either Sir Thomas Myles or Dr. Mayne should have yielded in the dispute. In our opinion, the stronger claim in this direction fell upon Dr. Mayne, and we think that, in refusing the use of the hospital theatre, he made an error of judgment. Even though, according to a somewhat sarcastic legal maxim, we run a risk by so doing, we will give our reasons for this conclusion. In the first place, the patient was a soldier, and Sir Thomas Myles, in view of the appointment he holds, could not divest himself of some measure of official responsibility for the adequate performance of the proposed operation. It is, of course, beyond question that he himself was abundantly competent to undertake the case, and his standing and repute as a hospital surgeon of experience and skill must have been perfectly well known to Dr. Mayne. For anything we know to the contrary Dr. Mayne also may have considerable surgical experience and skill, but it by no means follows that certainty on this point was possessed by Sir Thomas Myles, with whom, as we have already said, official responsibility for the proper care of the sick soldier in a greater or less degree undoubtedly rested. In the second place, we think Dr. Mayne might prudently have recognised that existing circumstances make the public judgment particularly sensitive in relation to any action which may appear harsh and unsympathetic towards a member of the Army on whose valour our dearest interests depend; and he might have felt sure that public opinion would be alienated by any action in which the interests of a suffering soldier were sacrificed for a scruple of professional dignity. It was open to him to have registered his protest at the time, and later to have raised the whole question for public and professional discussion. Had he taken such a course, we believe he would have commanded a considerable body of support. The decision of the committee, dismissing Dr. Mayne from his hospital position, we regard as wholly in excess of the necessities of the position, and we hope that the Local Government Board will not confirm it. Such a penalty is not the appropriate discipline for what was at most an error of judgment.

A large question raised by the dispute involves the relation between the civil hospitals and the military authorities. These hospitals have their duly appointed staffs, who are responsible for the proper treatment of the patients placed under their care. In the present juncture, some of these patients are sick and wounded soldiers. Is the authority of the ordinary staff to be superseded whenever there may arrive on the scene a gentleman boasting a military commission? If such is the official doctrine, it is time it were most strenuously challenged. Our civil hospitals are most freely at the disposal of the War Office in so far as they are needed, but if they are to be utilised in this fashion their domestic and internal management must be free from interference. The War Office can, of course, commandeer the hospitals, and thus can become fully responsible for their control. But it cannot leave the hospital committees and staffs to bear responsibility, and then, in occasional instances, supersede their authority by the invasion of a military official. The Longford incident illustrates the impossibility of such a position, and so far as Dr. Mayne's action is a protest against it we sympathise with him, though, for reasons already given, we think he was led into error in dealing with the circumstances of the individual case.

